# Survival differences in rheumatoid arthritis interstitial lung disease and idiopathic pulmonary fibrosis may be explained by delays in presentation: results from multivariate analysis in a monocentric UK study

**DOI:** 10.1007/s00296-023-05505-0

**Published:** 2023-12-12

**Authors:** Shirish Dubey, Felix Woodhead

**Affiliations:** 1 Department of Rheumatology, Oxford University Hospitals NHS FT, Windmill Road, Oxford, OX3 7HE UK; 2 Nuffield Department of Orthopaedics, Rheumatology and Musculoskeletal Sciences, Nuffield, University of Oxford, Windmill Road, Oxford, OX3 7LD UK; 3 Honorary Consultant Respiratory Physician, Glenfield Hospital, Leicester, LE3 9QP UK; 4 Avalyn Pharma, 701 Pike Street, Suite 1500, Seattle, WA 98101 USA

**Keywords:** RA-ILD, IPF, Pulmonary fibrosis, Mortality, Multi-variate, Progression

## Abstract

Rheumatoid arthritis-associated interstitial lung disease (RA-ILD) has a better prognosis compared to idiopathic pulmonary fibrosis (IPF). Recent data suggest that antifibrotics are effective in slowing progression across both groups. Hence, we designed this study to investigate the similarities and differences between these groups of patients. This is a retrospective cohort study examining baseline data, progression and outcomes in patients with RA-ILD and IPF prior to antifibrotic use in the Coventry ILD database. Ethics approval was obtained from the University Hospital Coventry and Warwickshire NHS Trust. Statistical analysis was performed using R software and Cox’s proportional hazards technique was used for survival analysis. We identified 131 cases, including 49 patients with IPF, 34 patients with RA-ILD and 48 patients with other forms of idiopathic interstitial pneumonia. At baseline, there were significant differences in the groups with RA-ILD patients being significantly younger (65.7 vs 72.4 years), had preserved lung volumes (FVC 95% vs 84.7%) and higher gas transfer (61.5% vs 48.2%) compared to IPF patients. 5-year survival was better for RA-ILD compared to IPF (87.5% vs 40.4%, *p* = 0.0042). Univariate analysis revealed gas transfer, FVC, age, sex and phenotype (IPF or RA-ILD) were all significant predictors, but multivariate analysis revealed that gas transfer and age were both significantly associated with prognosis, whereas sex, FVC or phenotype were not significant. This study suggests that the difference between RA-ILD and IPF prognosis may be due to demographics and early diagnosis rather than the diseases behaving differently. This has important management implications.

**Supplementary Information:**

The online version contains supplementary material available at 10.1007/s00296-023-05505-0.

## Introduction

Rheumatoid arthritis (RA) is a common autoimmune condition characterised by symmetrical inflammatory small joint polyarthropathy and loss of function. Systemic manifestations of RA including interstitial lung disease (ILD) are relatively common and thought to occur in up to 40% of patients [1]. ILD is associated with significant increase in morbidity and mortality in RA compared to patients who do not have ILD [2, 3]. ILD can have different patterns and the classification (and prognosis) of ILD is based on findings on the basis of radiological patterns as seen on computed tomography (CT) scans with the subtypes being usual interstitial pneumonia (UIP), non-specific interstitial pneumonia (NSIP), organising pneumonia (OP), desquamative interstitial pneumonia (DIP), respiratory bronchiolitis (RB-ILD) and diffuse alveolar damage (DAD) [4]. Apart from RA and other autoimmune conditions, ILD may be associated with exposure to inorganic or organic particles or to drugs. When no such association occurs, it is known as idiopathic interstitial pneumonia [5]. In 2000, following data showing an especially poor prognosis amongst patients with IIP with the pulmonary histopathology of usual interstitial pneumonia (UIP), idiopathic pulmonary fibrosis (IPF) were specifically defined as IIP with a UIP pattern on biopsy [6]. Over the following years, international consensus statements have refined the radiological appearances allowing a diagnosis of IPF without biopsy [7]. IPF has a very poor prognosis, with median survival of 3–5 years [6]. Whereas immunosuppression is widely used to treat RA and other autoimmune conditions, triple therapy with Prednisolone, azathioprine and N-acetyl cysteine was found to increase mortality in IPF [8]. This led to the development of antifibrotic agents such as pirfenidone [9] and nintedanib [10] which have been shown to reduce the rate of decline in forced vital capacity (FVC) in IPF and have become the standard of care [11].

There is some evidence to suggest improvement in mortality trends in rheumatoid arthritis-associated ILD (RA-ILD) over the last couple of decades and data from the Early Rheumatoid Arthritis Network (ERAN) suggest a better prognosis compared to previous datasets [12, 13]. There are also studies supporting a better prognosis for RA-ILD compared to IPF [14, 15]. UIP pattern is typically seen in both conditions whilst idiopathic interstitial pneumonia that is not IPF (IIP–not IPF) also has a better prognosis [6, 16].

Although ILD is well recognised in patients with RA, it is often picked up due to minor symptoms or on screening examinations [17]. Very few studies have directly compared RA-ILD with IPF, and there remains uncertainty about whether survival benefits correlate with pathophysiology [18]. Some recent studies have looked at progression in RA-ILD and a study on ‘early’ IPF showed slower rates of progression compared to more established disease [19, 20]. Historically there were no specific treatments for RA-ILD and recently antifibrotic drugs have been studied. The TRAIL1 study which directly examined pirfenidone vs placebo in RA-ILD closed recruitment prematurely due to the COVID-19 pandemic and its primary endpoint was negative, although a secondary endpoint suggested pirfenidone may have some efficacy at slowing FVC decline in RA-ILD [21]. RA-ILD was also one of the disease categories comprising ‘progressive fibrosing ILD’ (PFILD) in the INBUILD study [22, 23] and showed that nintedanib slowed FVC decline in these subjects.

To investigate differences between RA-ILD and IPF, we decided to conduct this study based on routinely collected historical data from University Hospital Coventry and Warwickshire NHS Trust (UHCW) for patients with ILD. Since antifibrotics were only available for IPF, we were concerned about the bias this would introduce. Hence, we decided to restrict the inclusion prior to the widespread use of antifibrotics in ILD care (pirfenidone was the first antifibrotic and this became available within National Health Service (NHS) following the National Institute for Health and Care Excellence Technology Appraisal (NICE TA) in 2013) [24] and compared baseline demographics, clinical and survival data.

## Methods

This is a retrospective cohort study conducted through the Coventry ILD database. The Coventry ILD database was set up in 2010 and all patients with interstitial lung disease were included in this including rheumatological and non-rheumatological ILDs. Patients that were suspected to have ILD were added to this database and all patients were discussed in the ILD MDT which comprised at least one chest physician, chest radiologist, histopathologist, respiratory nurse specialist and usually a rheumatologist. Patients were seen in the ILD clinics and patients with rheumatological ILDs were managed collaboratively by the rheumatology and respiratory teams through close links and regular combined clinics. We have retrospectively looked at this database to analyse progression in patients with RA-ILD vs idiopathic interstitial pneumonia (IIP) including IPF. Inclusion criteria included patients with definite ILD of rheumatological or other aetiologies who had not been treated with antifibrotics. Patients had to be followed-up locally so that serial data on clinical, physiological and other parameters were available. Other connective tissue disorders like systemic lupus erythematosus (SLE), scleroderma, myositis, mixed connective tissue disorder (MCTD) and overlap syndromes were excluded from this analysis. Similarly, patients with sarcoidosis or another defined respiratory or systemic aetiology for ILD were excluded. Data on disease-modifying anti-rheumatic drugs (DMARDs) including conventional synthetic (csDMARDs) agents such as Methotrexate and biological agents (bDMARDs) were also collected through the electronic patient records in the Trust.

ILD was classified on the basis of the American Thoracic Society/European Respiratory Society (ATS/ERS) criteria as per discussion in the ILD multidisciplinary team meeting (MDT) [6]. For this study, we were interested in comparing the outcomes of ILD in RA vs IPF and other types of IIP which did not meet ATS/ERS criteria for IPF. We included all available data for serial lung function tests (PFTs). Earliest PFT was from 31st July 2007. Latest initial PFT was from 19th October 2012. Data were anonymised prior to extraction, and only patients not treated with antifibrotics were included in this study.

Statistical analysis

Statistics was performed using ‘R’, an open-source statistics package [25]. A *p* value of < 0.05 was considered significant. Differences in continuous variables were assessed with Kruskal–Wallis test. Survival analyses were performed with Cox’s proportional hazards technique in both univariate and multivariate analysis.

Ethical approval was obtained from the GafREC committee of research, development and innovation department of University Hospital Coventry and Warwickshire NHS Trust—approval number GF 0265 dated 25th June 2018. No funding was available for this study.

## Results

We identified 131 cases who fulfilled the inclusion criteria and did not meet any of the exclusion criteria. These included 49 patients with IPF, 34 patients with RA-ILD and 48 patients had other forms of IIP.

### Demographics

Table 1 illustrates the baseline patient demographics. As expected, IPF patients were more likely to be male (36 males, 13 females) whereas with RA-ILD females formed the majority (12 males, 22 females). In the other IIP group, males were more common as well (29 males, 19 females). The majority of patients in all the groups had a background smoking history (current or ex-smokers) with 38 patients being non-smokers, 17 being current smokers and 76 being ex-smokers. There were 12 non-smokers in the IPF group, 19 in the other IIP group and 7 in the RA-ILD group, these differences were not significant. Duration of follow-up (FU) varied but median FU was 41 months.

**Table 1:** Baseline characteristics demonstrating that RA patients have higher FVC at presentation

Category	IPF mean (SD)	IIP–not IPF mean (SD)	RA-ILD mean (SD)	*p* value
Number	49	48	34	
Age	72.4 y (9.1)	70.8 y (9.6)	65.7 y (9.6)	**0.006**
FEV1	84.1% (17.1)	80.6% (20.0)	88.9% (17.7)	0.133
FVC	84.7% (20.6)	84.0% (19.3)	95.0% (18.1)	**0.026**
TLco	48.2% (14.6)	52.2% (18.1)	61.5% (17.1)	**0.002**

Statistically significant *p* values highlighted in bold

*SD* standard deviation, *y* years

There were several differences between the cohorts as expected. The RA-ILD patients were younger and had higher forced vital capacity (FVC) and pulmonary gas transfer (TLco) than those with either IPF or other types of IIP. Within the RA-ILD cohort, only 3 patients had baseline TLco lower than 50% (9%), whilst the IPF cohort had 27 patients (54%) with TLco lower than 50%. For FVC, 13 patients (30%) with IPF had values of < 70% at baseline whilst 4 patients (12%) with RA-ILD had FVC < 70%. There was wide distribution in the baseline lung functions as some patients having supra normal lung volumes, the high standard deviation in all three groups in Table 1 reflect this.

### Survival analysis

**Fig. 1:**
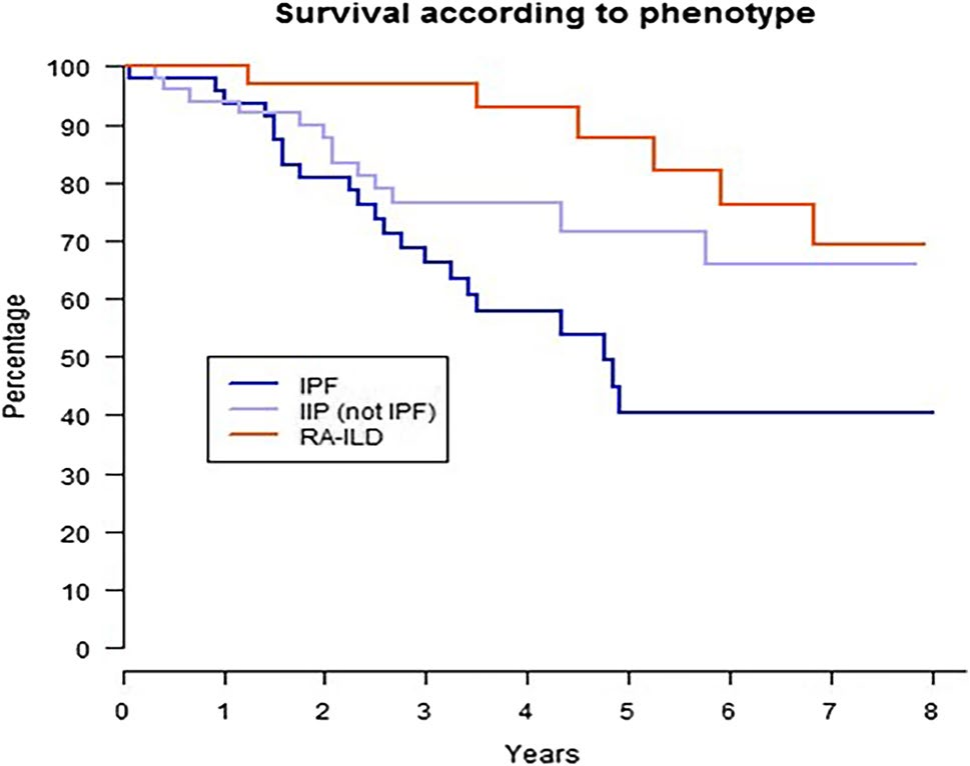
Survival rates for RA-ILD, IIP and IPF

We performed Kaplan–Meier analysis to assess survival differences (Fig. 1). Survival rates were very different in the three groups as illustrated by the table below. 5-year survival was 40.4% at 5 years for IPF; 87.5% at 5 years for RA-ILD and 71% at 5 years for IIP (not IPF), these differences are statistically significant (*p* = 0.0042).

### Univariate analysis

We performed outcome analysis using both univariate and multivariate models using Cox’s proportional hazard model. Predictors by univariate analysis are shown in Table 2. This showed that TL_CO_, FVC, age, sex and a diagnosis of either IPF or RA-ILD were all significant predictors.

**Table 2:** Univariate analysis of survival data

Factor	Hazard ratio (HR)	Lower 95%	Upper 95%	*p* value
TL_CO_	0.959	0.941	0.977	**2 × 10**^**−5**^
FVC	0.976	0.962	0.991	**0.002**
FEV1	0.989	0.975	1.004	0.141
Age	1.044	1.013	1.076	**0.005**
Sex	2.043	1.15	3.632	**0.015**
IPF	2.291	1.329	3.952	**0.003**
IIP (not IPF)	0.901	0.516	1.574	0.715
RA-ILD	0.363	0.177	0.746	**0.006**

Statistically significant *p* values highlighted in bold

### Multivariate analysis

All factors showing significant univariate association with mortality were sequentially added to a multivariate model, starting with the most significant (TLco). The addition of variables continued until one stopped showing addition significance. Gas transfer and age were both significantly associated with prognosis, whereas adding FVC or the phenotype (IPF or RA-ILD) provided no additional prognostic information (Table 3).

**Table 3:** Multivariate analysis of survival data

Model	Factor	*p *value
TL_co_ and age and IPF	TL_co_	**5 × 10**^**−5**^
	Age	**0.006**
	IPF	0.09
TL_co_ and age and RA-ILD	TL_co_	**8 × 10**^−**5**^
	Age	**0.009**
	RA-ILD	0.24

Statistically significant values highlighted in bold

### Rheumatological treatments

Rheumatological treatments for RA-ILD patients included Prednisolone, Methotrexate, Leflunomide, Hydroxychloroquine, Sulfasalazine, Cyclophosphamide and Mycophenolate mofetil among the conventional synthetic DMARDs and Rituximab, Adalimumab and Abatacept amongst the biologic DMARDs. Associations between rheumatological treatments and survival were analysed. Numbers were small and no significant associations were seen.

## Discussion

The key findings from our study are that age and gas transfer were significantly associated with outcomes and diagnosis of RA-ILD or IPF did not make a difference. This suggests that the survival differences between RA-ILD and IPF may be due to earlier diagnosis rather than due to inherent differences in the underlying aetiology of the illness. Previous studies suggested that RA-ILD had a better outcome than IPF and our study also suggests the same [14, 18]. However, multivariate analysis suggests that the apparent difference may be related to the fact that patients with RA-ILD were younger and had better baseline lung function at first diagnosis suggesting that early diagnosis was the primary reason for disparate outcomes. The main determinants of survival between subjects with these ILDs were age and lung gas transfer. Whilst RA-ILD and IPF have been investigated previously, we do not believe that this kind of analysis has been performed previously and this study demonstrates some interesting findings that have significant clinical impact.

There are several reasons why RA-ILD might be diagnosed earlier—rheumatologists would often ask for chest symptoms such as cough, shortness of breath and auscultate the chest in new patients with inflammatory arthritis. Baseline chest X-rays are often done prior to DMARD therapy and PFT is also recommended for some patients [26, 27]. In practise, some clinicians would routinely request PFT at the time of therapy initiation in patients with RA; hence, ILD if present is likely to be picked much earlier. This has been an established practise for patients with smoking history which is also now recognised as a risk factor for development of ILD [28]. Also, patients who have chest and joint symptoms could take this more seriously and might be more likely to approach the General Practitioner (GP) quicker and may be considered sicker and get referred earlier from primary care. Both age and pulmonary diffusion are likely to be affected by early diagnosis and would be better with early diagnosis. An interesting Japanese study in patients with IPF with no physiological impairment revealed that the rate of loss of lung volume in the first was only 83 mls, contrasting with the more established patients with IPF who progress at approximately 150–200 mls volume loss [20, 29].

Previous studies have shown conflicting results on the impact of the pattern of lung involvement on mortality with some studies suggesting that UIP pattern correlates with worse prognosis and some not finding this association [19]. We did not differentiate between UIP and non-specific interstitial pneumonia (NSIP) in the RA cohort within our study, as we have noted that some patients who start off with NSIP pattern develop significant fibrotic change over time. Interestingly, a recent meta-analysis did find a difference between NSIP and UIP patterns but also concluded that ‘recent studies emphasise the importance of pulmonary physiology and the extent of lung involvement as significant predictors of mortality rather than the pattern of RA-ILD’ [30].

Our study did not demonstrate any impact of DMARDs either conventional or biological agents. It is interesting that a few patients received Cyclophosphamide for progressive lung disease in the absence of joint inflammation to justify a biological agent. At the time, NICE criteria for eligibility for a biological agent were failure of 2 csDMARDs and disease activity score in the form of DAS 28 score of > 5.1 on 2 occasions at least 4 weeks apart. [31]. However, the number of patients on individual DMARDs (both cs and biological) become small, and it is difficult to draw any meaningful conclusions on this aspect. Methotrexate has more recently gained a lot of attention and a large study with more than 1000 patients suggested a lower risk of subsequently developing ILD in RA patients treated with Methotrexate [32]. A much bigger prospective cohort study from multiple centres is needed to fully understand the impact of rheumatological treatments on development and progression of RA-ILD. This is suggesting that there is window of opportunity in early disease (or perhaps pre-clinical disease in predisposed individuals such as individuals with genetic mutations) that may be responsive to therapeutic options such as immunomodulation. Once fibrotic disease is established, immunological therapies have a limited role from the lung perspective. The validity of this concept for IPF and other forms of IIP needs testing and specific targeted treatments may be of value in selected patients with early disease or at risk of disease.

There is a school of thought that UIP from any aetiology should be considered the same as a diagnostic entity which would include RA-UIP, hypersensitivity pneumonitis, etc. [33]. This would support the argument that RA-ILD and IPF should be considered as similar conditions. We do not know whether antifibrotics would be equally effective in RA-ILD as compared to IPF patients. This needs formal assessment in controlled studies; however, the similarities in clinical patterns and data from INBUILD [23] and SCENCIS [34] studies would suggest that antifibrotics should be equally effective, when tolerated—tolerance does appear to be lower. However, antifibrotics are only slowing progression and not stopping progression, and do not have an effect on the underlying immunological mechanisms causing disease; hence, there continues to be an unmet need for agents that would be more effective in treatment of ILDs. There is also a role for drugs which can be administered differently such as inhaled antifibrotics.

### Limitations

This is a retrospective single-centre cohort study with limited numbers and studies of this design have a number of limitations that apply to this study as well. The data are relatively old but provides us an opportunity to look at patients who have not been on antifibrotics which would be a confounder in this case.

## Conclusion

This single-centre study found that age and diffusion capacity at presentation are the best predictors of outcome and did not find that the diagnosis of RA-ILD or IPF was significant. Although IPF has a shorter life expectancy compared to RA-ILD, multivariate analysis in our study suggests that this may be due to delays in diagnosis rather than being a different phenotype.

### Supplementary Information

Below is the link to the electronic supplementary material.

Supplementary file1 (DOCX 15 KB)
