# Supplementary material for: Survival differences in rheumatoid arthritis interstitial lung disease and idiopathic pulmonary fibrosis may be explained by delays in presentation: results from multivariate analysis in a monocentric UK study
Source: Rheumatol Int. 2023 Dec 12;44(1):99–105. doi: 10.1007/s00296-023-05505-0 (PMC10766668; doi:10.1007/s00296-023-05505-0)
Supplement: Supplementary file 1 — Supplementary file1 (DOCX 15 KB) [file 296_2023_5505_MOESM1_ESM.docx]

Supplementary data:

| Drug | Died | Not Died | HR | low CI | high CI | p |
| --- | --- | --- | --- | --- | --- | --- |
| csDMARDs |  |  |  |  |  |  |
| Cyclophosphamide | 0 | 3 | 1.35e-08 | 0 | Inf | 0.999 |
| Hydroxychloroquine | 1 | 4 | 1.19 | 0.147 | 9.58 | 0.873 |
| Leflunomide | 0 | 3 | 1.34e-08 | 0 | Inf | 0.999 |
| Methotrexate | 5 | 16 | 1.13 | 0.3 | 4.24 | 0.858 |
| Mycophenolate Mofetil | 0 | 3 | 1.33e-08 | 0 | Inf | 0.999 |
| Prednisolone | 3 | 8 | 0.999 | 0.249 | 4 | 0.999 |
| Sulfasalazine | 3 | 13 | 1.04 | 0.253 | 4.27 | 0.957 |
| bDMARDs |  |  |  |  |  |  |
| Abatacept | 0 | 3 | 1.3e-08 | 0 | Inf | 0.998 |
| Adalimumab | 1 | 2 | 2.47 | 0.509 | 12 | 0.262 |
| Etanercept | 0 | 0 | na | na | na | na |
| Infliximab | 0 | 0 | na | na | na | na |
| Rituximab | 4 | 9 | 0.962 | 0.253 | 3.65 | 0.954 |
| Tocilizumab | 0 | 0 | na | na | na | na |
|  |  |  |  |  |  |  |

Table 4: Association of rheumatological treatments including cs and bDMARDs with outcomes in RA-ILD.
